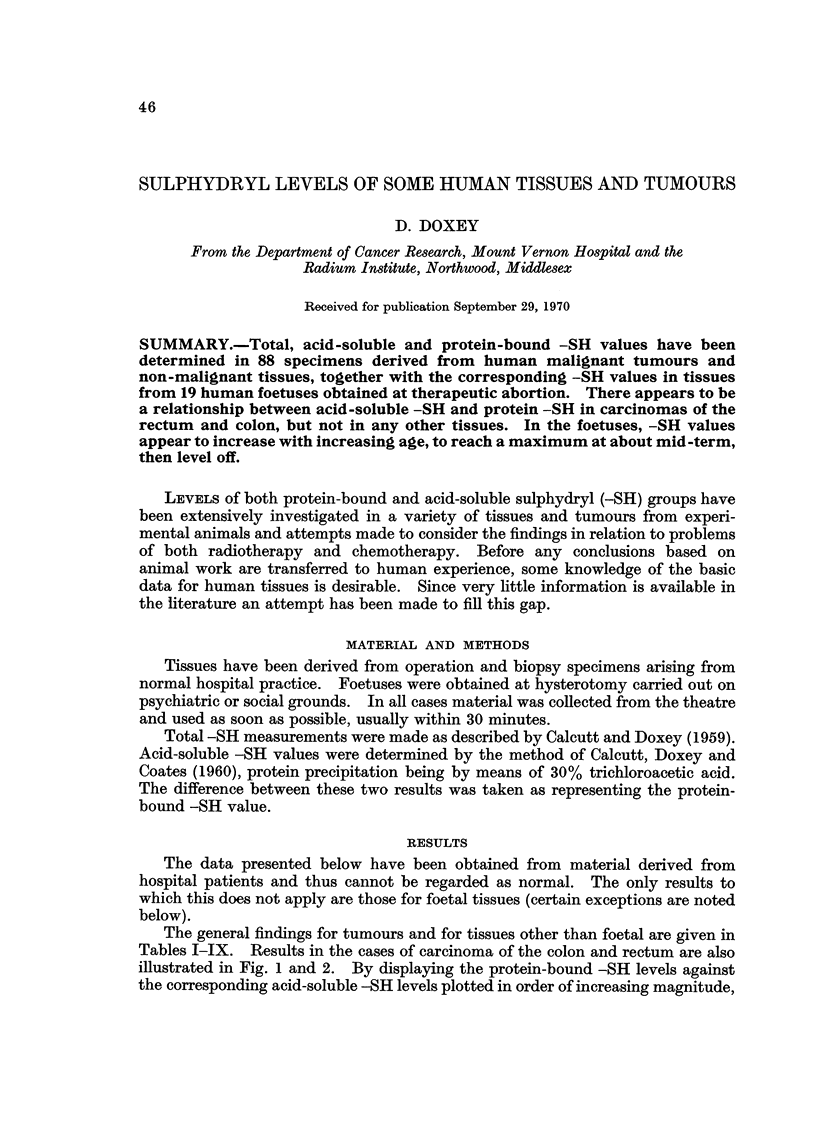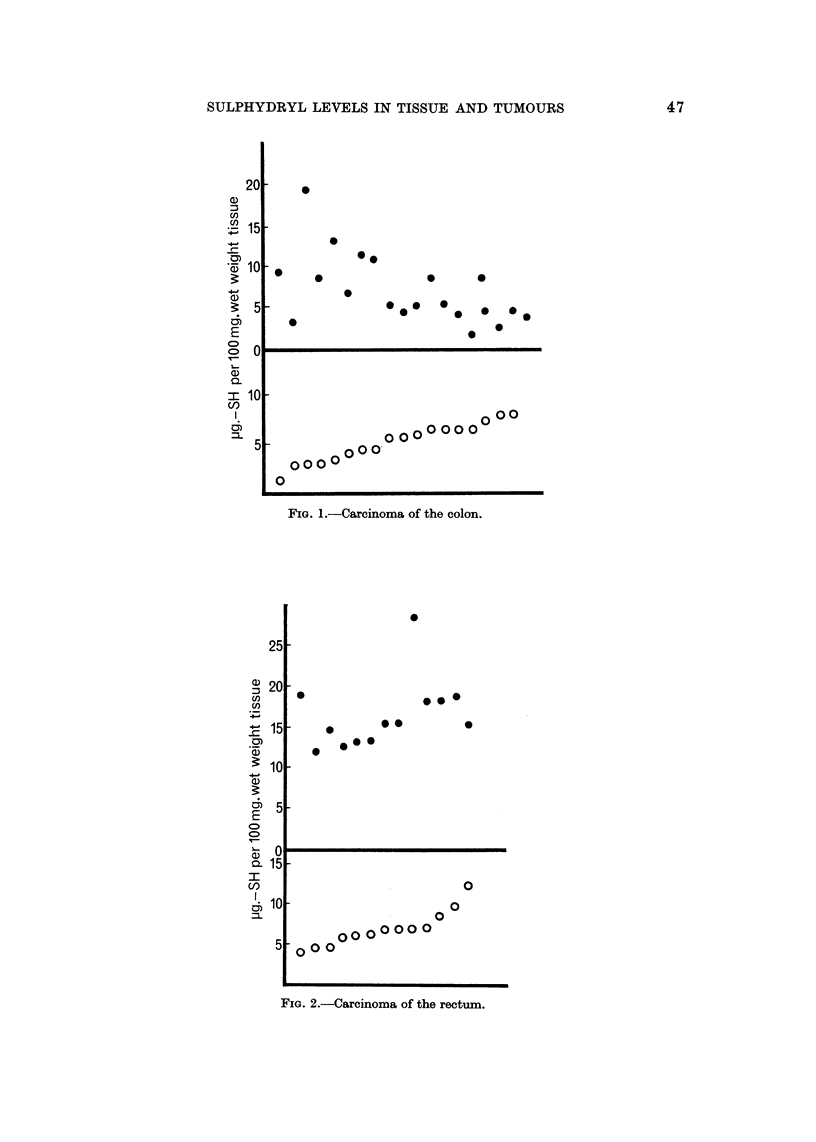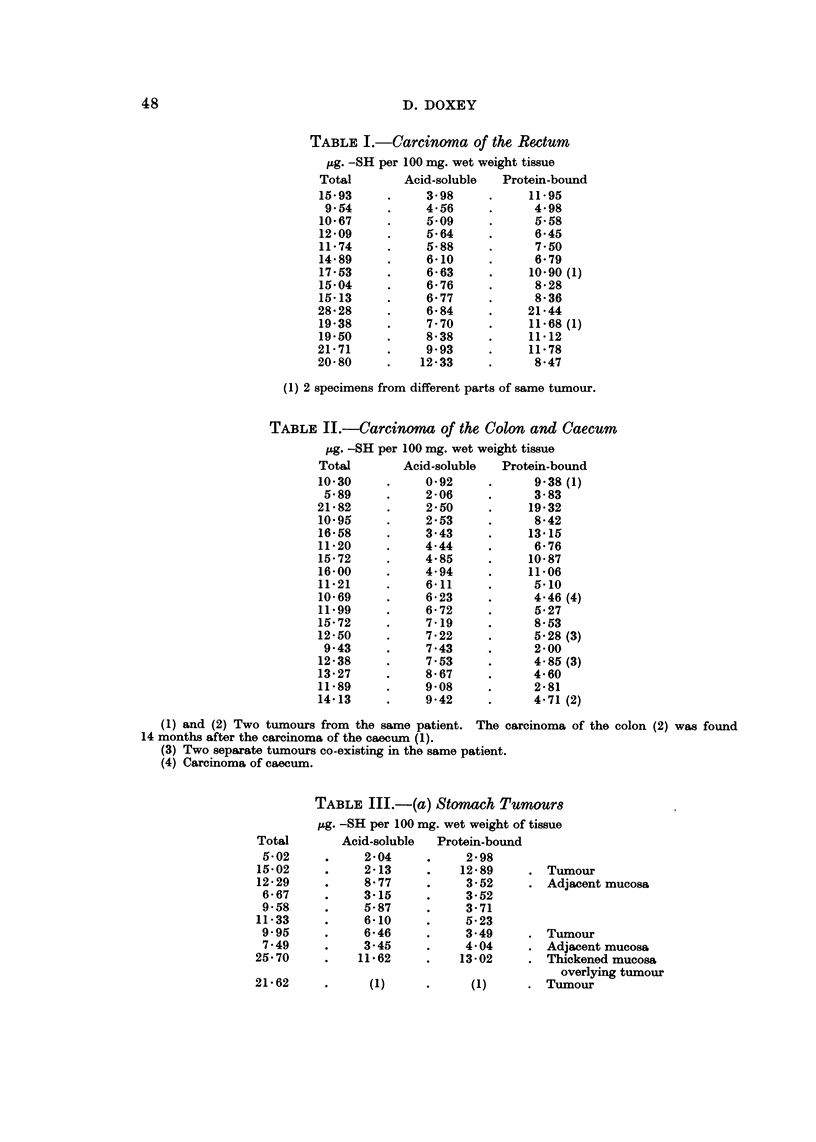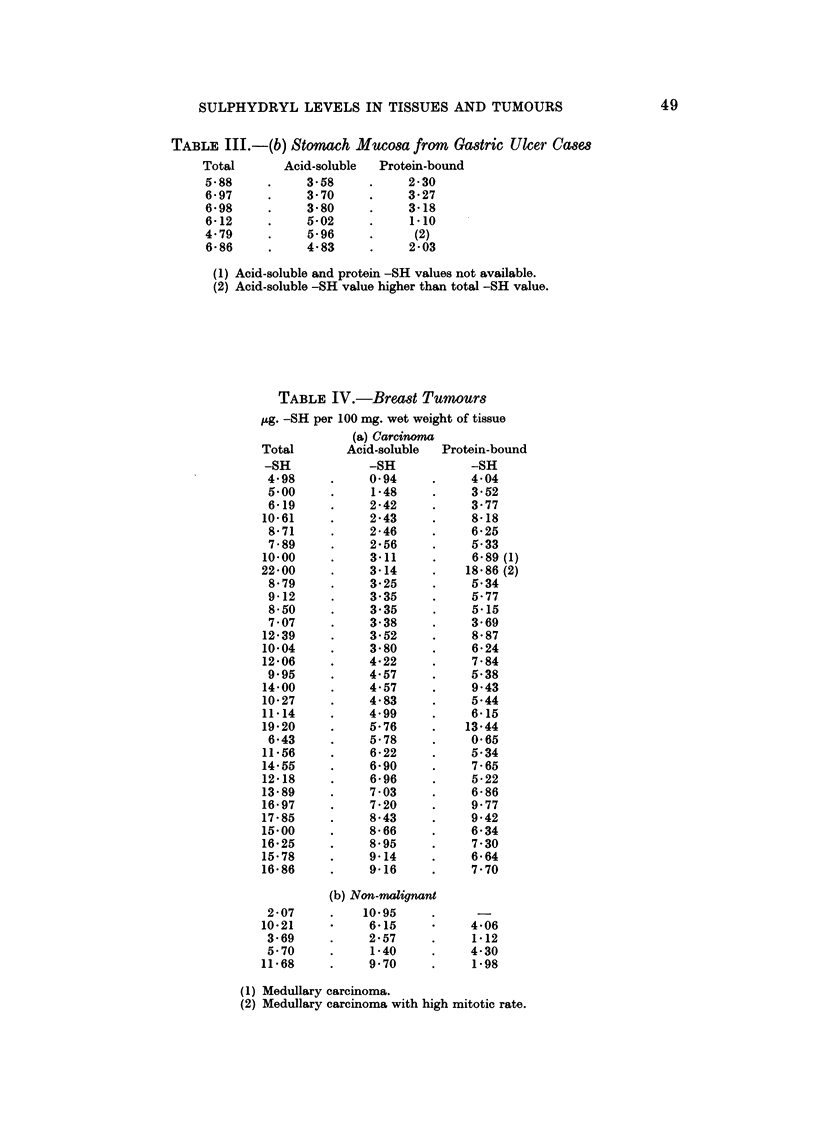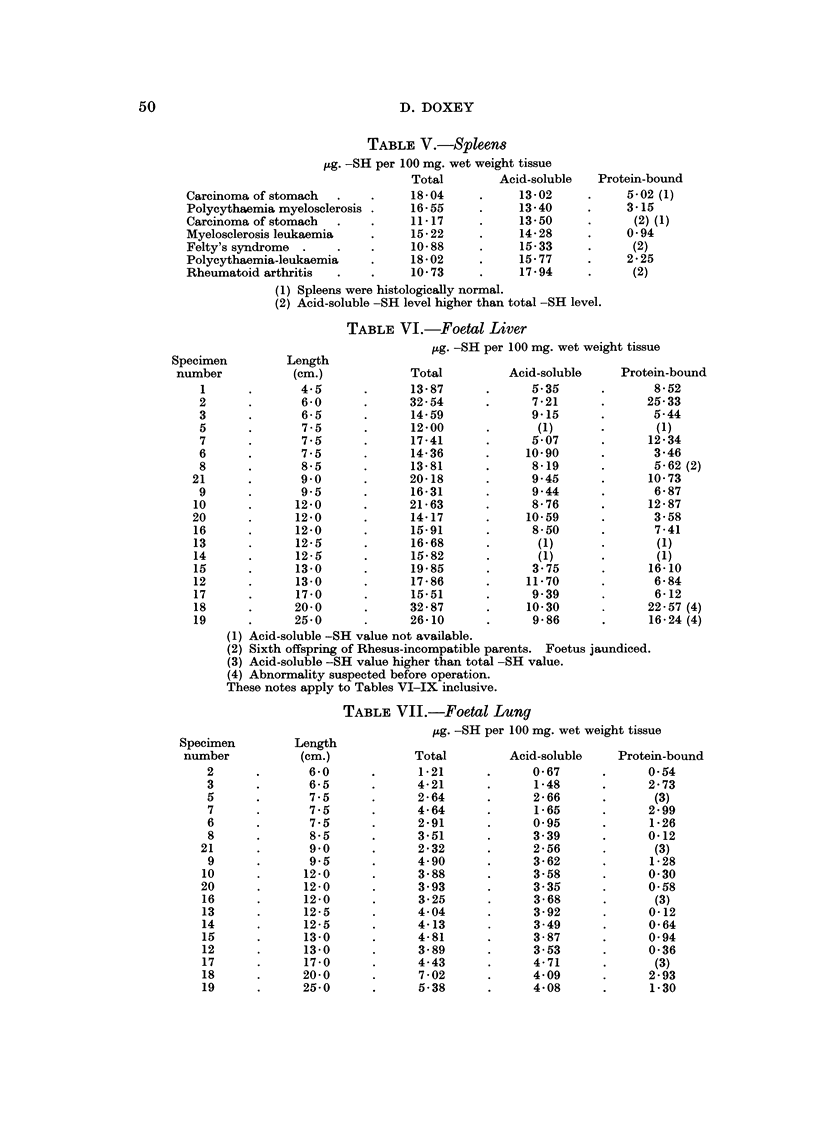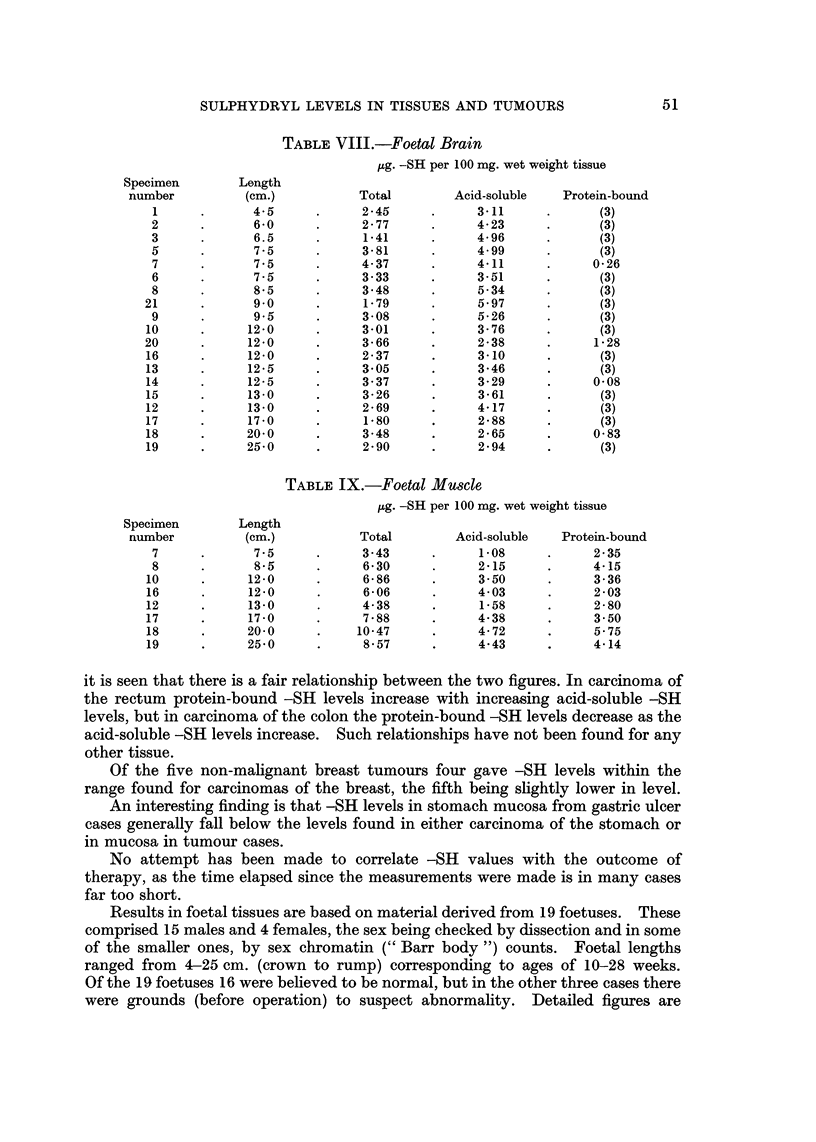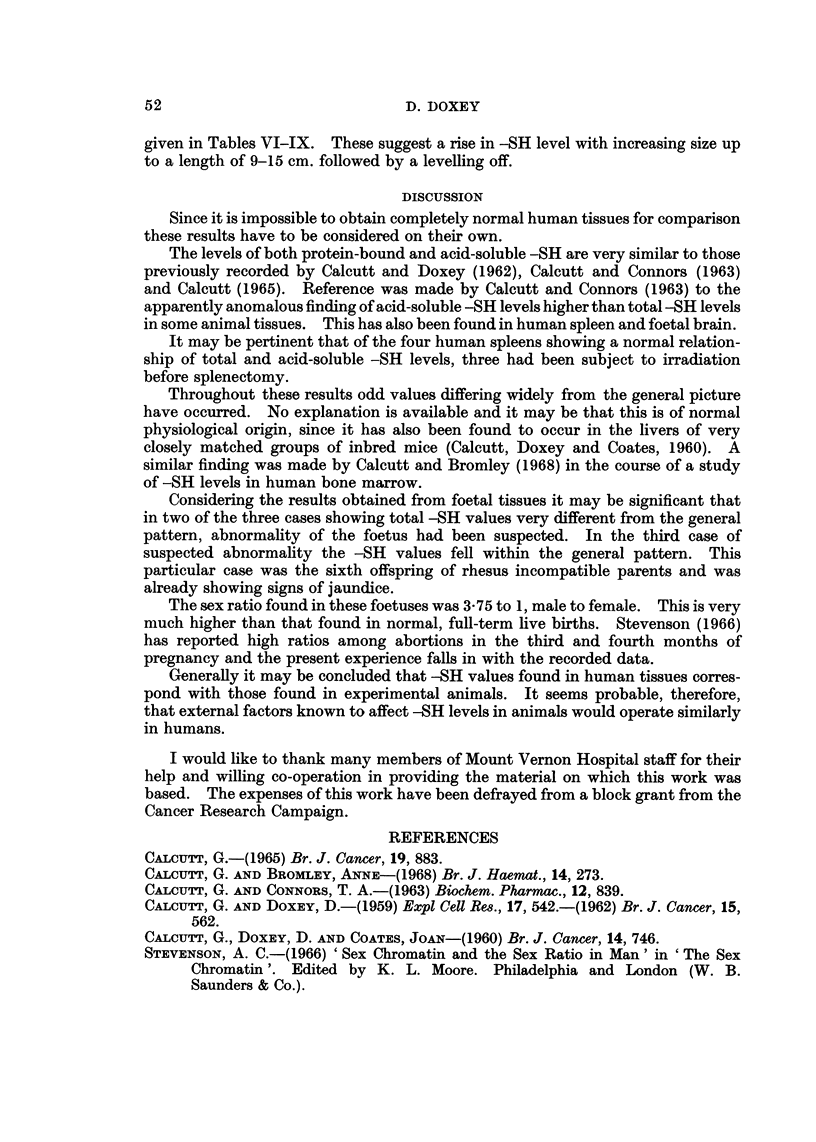# Sulphydryl Levels of Some Human Tissues and Tumours

**DOI:** 10.1038/bjc.1971.7

**Published:** 1971-03

**Authors:** D. Doxey

## Abstract

Total, acid-soluble and protein-bound -SH values have been determined in 88 specimens derived from human malignant tumours and non-malignant tissues, together with the corresponding -SH values in tissues from 19 human foetuses obtained at therapeutic abortion. There appears to be a relationship between acid-soluble -SH and protein -SH in carcinomas of the rectum and colon, but not in any other tissues. In the foetuses, -SH values appear to increase with increasing age, to reach a maximum at about mid-term, then level off.


### SULPHYDRYL LEVELS OF SOME HUMAN TISSUES AND TUMOURS

D. DOXEY

*From the Department of Cancer Research, Mount Vernon Hospital and the Radium Institute, Northwood, Middlesex*

Received for publication September 29, 1970

**SUMMARY.—Total, acid-soluble and protein-bound –SH values have been determined in 88 specimens derived from human malignant tumours and non-malignant tissues, together with the corresponding –SH values in tissues from 19 human foetuses obtained at therapeutic abortion. There appears to be a relationship between acid-soluble –SH and protein –SH in carcinomas of the rectum and colon, but not in any other tissues. In the foetuses, –SH values appear to increase with increasing age, to reach a maximum at about mid-term, then level off.**

LEVELS of both protein-bound and acid-soluble sulphydryl (–SH) groups have been extensively investigated in a variety of tissues and tumours from experimental animals and attempts made to consider the findings in relation to problems of both radiotherapy and chemotherapy. Before any conclusions based on animal work are transferred to human experience, some knowledge of the basic data for human tissues is desirable. Since very little information is available in the literature an attempt has been made to fill this gap.

### MATERIAL AND METHODS

Tissues have been derived from operation and biopsy specimens arising from normal hospital practice. Foetuses were obtained at hysterotomy carried out on psychiatric or social grounds. In all cases material was collected from the theatre and used as soon as possible, usually within 30 minutes.

Total –SH measurements were made as described by Calcutt and Doxey (1959). Acid-soluble –SH values were determined by the method of Calcutt, Doxey and Coates (1960), protein precipitation being by means of 30% trichloroacetic acid. The difference between these two results was taken as representing the protein-bound –SH value.

### RESULTS

The data presented below have been obtained from material derived from hospital patients and thus cannot be regarded as normal. The only results to which this does not apply are those for foetal tissues (certain exceptions are noted below).

The general findings for tumours and for tissues other than foetal are given in Tables I–IX. Results in the cases of carcinoma of the colon and rectum are also illustrated in Fig. 1 and 2. By displaying the protein-bound –SH levels against the corresponding acid-soluble –SH levels plotted in order of increasing magnitude,

FIG. 1.—Carcinoma of the colon.

FIG. 2.—Carcinoma of the rectum.

TABLE I.—*Carcinoma of the Rectum*

μg. –SH per 100 mg. wet weight tissue

| Total | Acid-soluble | Protein-bound |
|---|---|---|
| 15·93 | 3·98 | 11·95 |
| 9·54 | 4·56 | 4·98 |
| 10·67 | 5·09 | 5·58 |
| 12·09 | 5·64 | 6·45 |
| 11·74 | 5·88 | 7·50 |
| 14·89 | 6·10 | 6·79 |
| 17·53 | 6·63 | 10·90 (1) |
| 15·04 | 6·76 | 8·28 |
| 15·13 | 6·77 | 8·36 |
| 28·28 | 6·84 | 21·44 |
| 19·38 | 7·70 | 11·68 (1) |
| 19·50 | 8·38 | 11·12 |
| 21·71 | 9·93 | 11·78 |
| 20·80 | 12·33 | 8·47 |

(1) 2 specimens from different parts of same tumour.

TABLE II.—*Carcinoma of the Colon and Caecum*

μg. –SH per 100 mg. wet weight tissue

| Total | Acid-soluble | Protein-bound |
|---|---|---|
| 10·30 | 0·92 | 9·38 (1) |
| 5·89 | 2·06 | 3·83 |
| 21·82 | 2·50 | 19·32 |
| 10·95 | 2·53 | 8·42 |
| 16·58 | 3·43 | 13·15 |
| 11·20 | 4·44 | 6·76 |
| 15·72 | 4·85 | 10·87 |
| 16·00 | 4·94 | 11·06 |
| 11·21 | 6·11 | 5·10 |
| 10·69 | 6·23 | 4·46 (4) |
| 11·99 | 6·72 | 5·27 |
| 15·72 | 7·19 | 8·53 |
| 12·50 | 7·22 | 5·28 (3) |
| 9·43 | 7·43 | 2·00 |
| 12·38 | 7·53 | 4·85 (3) |
| 13·27 | 8·67 | 4·60 |
| 11·89 | 9·08 | 2·81 |
| 14·13 | 9·42 | 4·71 (2) |

(1) and (2) Two tumours from the same patient. The carcinoma of the colon (2) was found 14 months after the carcinoma of the caecum (1).

(3) Two separate tumours co-existing in the same patient.

(4) Carcinoma of caecum.

TABLE III.—(*a*) *Stomach Tumours*

μg. –SH per 100 mg. wet weight of tissue

| Total | Acid-soluble | Protein-bound | |
|---|---|---|---|
| 5·02 | 2·04 | 2·98 | |
| 15·02 | 2·13 | 12·89 | Tumour |
| 12·29 | 8·77 | 3·52 | Adjacent mucosa |
| 6·67 | 3·15 | 3·52 | |
| 9·58 | 5·87 | 3·71 | |
| 11·33 | 6·10 | 5·23 | |
| 9·95 | 6·46 | 3·49 | Tumour |
| 7·49 | 3·45 | 4·04 | Adjacent mucosa |
| 25·70 | 11·62 | 13·02 | Thickened mucosa overlying tumour |
| 21·62 | (1) | (1) | Tumour |

TABLE III.—(*b*) *Stomach Mucosa from Gastric Ulcer Cases*

| Total | Acid-soluble | Protein-bound |
|---|---|---|
| 5·88 | 3·58 | 2·30 |
| 6·97 | 3·70 | 3·27 |
| 6·98 | 3·80 | 3·18 |
| 6·12 | 5·02 | 1·10 |
| 4·79 | 5·96 | (2) |
| 6·86 | 4·83 | 2·03 |

(1) Acid-soluble and protein –SH values not available.
(2) Acid-soluble –SH value higher than total –SH value.

TABLE IV.—*Breast Tumours*

μg. –SH per 100 mg. wet weight of tissue

(a) *Carcinoma*

| Total –SH | Acid-soluble –SH | Protein-bound –SH |
|---|---|---|
| 4·98 | 0·94 | 4·04 |
| 5·00 | 1·48 | 3·52 |
| 6·19 | 2·42 | 3·77 |
| 10·61 | 2·43 | 8·18 |
| 8·71 | 2·46 | 6·25 |
| 7·89 | 2·56 | 5·33 |
| 10·00 | 3·11 | 6·89 (1) |
| 22·00 | 3·14 | 18·86 (2) |
| 8·79 | 3·25 | 5·34 |
| 9·12 | 3·35 | 5·77 |
| 8·50 | 3·35 | 5·15 |
| 7·07 | 3·38 | 3·69 |
| 12·39 | 3·52 | 8·87 |
| 10·04 | 3·80 | 6·24 |
| 12·06 | 4·22 | 7·84 |
| 9·95 | 4·57 | 5·38 |
| 14·00 | 4·57 | 9·43 |
| 10·27 | 4·83 | 5·44 |
| 11·14 | 4·99 | 6·15 |
| 19·20 | 5·76 | 13·44 |
| 6·43 | 5·78 | 0·65 |
| 11·56 | 6·22 | 5·34 |
| 14·55 | 6·90 | 7·65 |
| 12·18 | 6·96 | 5·22 |
| 13·89 | 7·03 | 6·86 |
| 16·97 | 7·20 | 9·77 |
| 17·85 | 8·43 | 9·42 |
| 15·00 | 8·66 | 6·34 |
| 16·25 | 8·95 | 7·30 |
| 15·78 | 9·14 | 6·64 |
| 16·86 | 9·16 | 7·70 |

(b) *Non-malignant*

| Total –SH | Acid-soluble –SH | Protein-bound –SH |
|---|---|---|
| 2·07 | 10·95 | — |
| 10·21 | 6·15 | 4·06 |
| 3·69 | 2·57 | 1·12 |
| 5·70 | 1·40 | 4·30 |
| 11·68 | 9·70 | 1·98 |

(1) Medullary carcinoma.
(2) Medullary carcinoma with high mitotic rate.

TABLE V.—*Spleens*

| | µg. –SH per 100 mg. wet weight tissue | | |
|---|---|---|---|
| | Total | Acid-soluble | Protein-bound |
| Carcinoma of stomach | 18·04 | 13·02 | 5·02 (1) |
| Polycythaemia myelosclerosis | 16·55 | 13·40 | 3·15 |
| Carcinoma of stomach | 11·17 | 13·50 | (2) (1) |
| Myelosclerosis leukaemia | 15·22 | 14·28 | 0·94 |
| Felty's syndrome | 10·88 | 15·33 | (2) |
| Polycythaemia-leukaemia | 18·02 | 15·77 | 2·25 |
| Rheumatoid arthritis | 10·73 | 17·94 | (2) |

(1) Spleens were histologically normal.
(2) Acid-soluble –SH level higher than total –SH level.

TABLE VI.—*Foetal Liver*

| Specimen number | Length (cm.) | µg. –SH per 100 mg. wet weight tissue: Total | Acid-soluble | Protein-bound |
|---|---|---|---|---|
| 1 | 4·5 | 13·87 | 5·35 | 8·52 |
| 2 | 6·0 | 32·54 | 7·21 | 25·33 |
| 3 | 6·5 | 14·59 | 9·15 | 5·44 |
| 5 | 7·5 | 12·00 | (1) | (1) |
| 7 | 7·5 | 17·41 | 5·07 | 12·34 |
| 6 | 7·5 | 14·36 | 10·90 | 3·46 |
| 8 | 8·5 | 13·81 | 8·19 | 5·62 (2) |
| 21 | 9·0 | 20·18 | 9·45 | 10·73 |
| 9 | 9·5 | 16·31 | 9·44 | 6·87 |
| 10 | 12·0 | 21·63 | 8·76 | 12·87 |
| 20 | 12·0 | 14·17 | 10·59 | 3·58 |
| 16 | 12·0 | 15·91 | 8·50 | 7·41 |
| 13 | 12·5 | 16·68 | (1) | (1) |
| 14 | 12·5 | 15·82 | (1) | (1) |
| 15 | 13·0 | 19·85 | 3·75 | 16·10 |
| 12 | 13·0 | 17·86 | 11·70 | 6·84 |
| 17 | 17·0 | 15·51 | 9·39 | 6·12 |
| 18 | 20·0 | 32·87 | 10·30 | 22·57 (4) |
| 19 | 25·0 | 26·10 | 9·86 | 16·24 (4) |

(1) Acid-soluble –SH value not available.
(2) Sixth offspring of Rhesus-incompatible parents. Foetus jaundiced.
(3) Acid-soluble –SH value higher than total –SH value.
(4) Abnormality suspected before operation.
These notes apply to Tables VI–IX inclusive.

TABLE VII.—*Foetal Lung*

| Specimen number | Length (cm.) | µg. –SH per 100 mg. wet weight tissue: Total | Acid-soluble | Protein-bound |
|---|---|---|---|---|
| 2 | 6·0 | 1·21 | 0·67 | 0·54 |
| 3 | 6·5 | 4·21 | 1·48 | 2·73 |
| 5 | 7·5 | 2·64 | 2·66 | (3) |
| 7 | 7·5 | 4·64 | 1·65 | 2·99 |
| 6 | 7·5 | 2·91 | 0·95 | 1·26 |
| 8 | 8·5 | 3·51 | 3·39 | 0·12 |
| 21 | 9·0 | 2·32 | 2·56 | (3) |
| 9 | 9·5 | 4·90 | 3·62 | 1·28 |
| 10 | 12·0 | 3·88 | 3·58 | 0·30 |
| 20 | 12·0 | 3·93 | 3·35 | 0·58 |
| 16 | 12·0 | 3·25 | 3·68 | (3) |
| 13 | 12·5 | 4·04 | 3·92 | 0·12 |
| 14 | 12·5 | 4·13 | 3·49 | 0·64 |
| 15 | 13·0 | 4·81 | 3·87 | 0·94 |
| 12 | 13·0 | 3·89 | 3·53 | 0·36 |
| 17 | 17·0 | 4·43 | 4·71 | (3) |
| 18 | 20·0 | 7·02 | 4·09 | 2·93 |
| 19 | 25·0 | 5·38 | 4·08 | 1·30 |

TABLE VIII.—*Foetal Brain*

| Specimen number | Length (cm.) | Total | Acid-soluble | Protein-bound |
|---|---|---|---|---|
| | | μg. –SH per 100 mg. wet weight tissue | | |
| 1 | 4·5 | 2·45 | 3·11 | (3) |
| 2 | 6·0 | 2·77 | 4·23 | (3) |
| 3 | 6.5 | 1·41 | 4·96 | (3) |
| 5 | 7·5 | 3·81 | 4·99 | (3) |
| 7 | 7·5 | 4·37 | 4·11 | 0·26 |
| 6 | 7·5 | 3·33 | 3·51 | (3) |
| 8 | 8·5 | 3·48 | 5·34 | (3) |
| 21 | 9·0 | 1·79 | 5·97 | (3) |
| 9 | 9·5 | 3·08 | 5·26 | (3) |
| 10 | 12·0 | 3·01 | 3·76 | (3) |
| 20 | 12·0 | 3·66 | 2·38 | 1·28 |
| 16 | 12·0 | 2·37 | 3·10 | (3) |
| 13 | 12·5 | 3·05 | 3·46 | (3) |
| 14 | 12·5 | 3·37 | 3·29 | 0·08 |
| 15 | 13·0 | 3·26 | 3·61 | (3) |
| 12 | 13·0 | 2·69 | 4·17 | (3) |
| 17 | 17·0 | 1·80 | 2·88 | (3) |
| 18 | 20·0 | 3·48 | 2·65 | 0·83 |
| 19 | 25·0 | 2·90 | 2·94 | (3) |

TABLE IX.—*Foetal Muscle*

| Specimen number | Length (cm.) | Total | Acid-soluble | Protein-bound |
|---|---|---|---|---|
| | | μg. –SH per 100 mg. wet weight tissue | | |
| 7 | 7·5 | 3·43 | 1·08 | 2·35 |
| 8 | 8·5 | 6·30 | 2·15 | 4·15 |
| 10 | 12·0 | 6·86 | 3·50 | 3·36 |
| 16 | 12·0 | 6·06 | 4·03 | 2·03 |
| 12 | 13·0 | 4·38 | 1·58 | 2·80 |
| 17 | 17·0 | 7·88 | 4·38 | 3·50 |
| 18 | 20·0 | 10·47 | 4·72 | 5·75 |
| 19 | 25·0 | 8·57 | 4·43 | 4·14 |

it is seen that there is a fair relationship between the two figures. In carcinoma of the rectum protein-bound –SH levels increase with increasing acid-soluble –SH levels, but in carcinoma of the colon the protein-bound –SH levels decrease as the acid-soluble –SH levels increase. Such relationships have not been found for any other tissue.

Of the five non-malignant breast tumours four gave –SH levels within the range found for carcinomas of the breast, the fifth being slightly lower in level.

An interesting finding is that –SH levels in stomach mucosa from gastric ulcer cases generally fall below the levels found in either carcinoma of the stomach or in mucosa in tumour cases.

No attempt has been made to correlate –SH values with the outcome of therapy, as the time elapsed since the measurements were made is in many cases far too short.

Results in foetal tissues are based on material derived from 19 foetuses. These comprised 15 males and 4 females, the sex being checked by dissection and in some of the smaller ones, by sex chromatin (" Barr body ") counts. Foetal lengths ranged from 4–25 cm. (crown to rump) corresponding to ages of 10–28 weeks. Of the 19 foetuses 16 were believed to be normal, but in the other three cases there were grounds (before operation) to suspect abnormality. Detailed figures are given in Tables VI–IX. These suggest a rise in –SH level with increasing size up to a length of 9–15 cm. followed by a levelling off.

### DISCUSSION

Since it is impossible to obtain completely normal human tissues for comparison these results have to be considered on their own.

The levels of both protein-bound and acid-soluble –SH are very similar to those previously recorded by Calcutt and Doxey (1962), Calcutt and Connors (1963) and Calcutt (1965). Reference was made by Calcutt and Connors (1963) to the apparently anomalous finding of acid-soluble –SH levels higher than total –SH levels in some animal tissues. This has also been found in human spleen and foetal brain.

It may be pertinent that of the four human spleens showing a normal relationship of total and acid-soluble –SH levels, three had been subject to irradiation before splenectomy.

Throughout these results odd values differing widely from the general picture have occurred. No explanation is available and it may be that this is of normal physiological origin, since it has also been found to occur in the livers of very closely matched groups of inbred mice (Calcutt, Doxey and Coates, 1960). A similar finding was made by Calcutt and Bromley (1968) in the course of a study of –SH levels in human bone marrow.

Considering the results obtained from foetal tissues it may be significant that in two of the three cases showing total –SH values very different from the general pattern, abnormality of the foetus had been suspected. In the third case of suspected abnormality the –SH values fell within the general pattern. This particular case was the sixth offspring of rhesus incompatible parents and was already showing signs of jaundice.

The sex ratio found in these foetuses was 3·75 to 1, male to female. This is very much higher than that found in normal, full-term live births. Stevenson (1966) has reported high ratios among abortions in the third and fourth months of pregnancy and the present experience falls in with the recorded data.

Generally it may be concluded that –SH values found in human tissues correspond with those found in experimental animals. It seems probable, therefore, that external factors known to affect –SH levels in animals would operate similarly in humans.

I would like to thank many members of Mount Vernon Hospital staff for their help and willing co-operation in providing the material on which this work was based. The expenses of this work have been defrayed from a block grant from the Cancer Research Campaign.

### REFERENCES

Calcutt, G.—(1965) *Br. J. Cancer*, **19**, 883.

Calcutt, G. and Bromley, Anne—(1968) *Br. J. Haemat.*, **14**, 273.

Calcutt, G. and Connors, T. A.—(1963) *Biochem. Pharmac.*, **12**, 839.

Calcutt, G. and Doxey, D.—(1959) *Expl Cell Res.*, **17**, 542.—(1962) *Br. J. Cancer*, **15**, 562.

Calcutt, G., Doxey, D. and Coates, Joan—(1960) *Br. J. Cancer*, **14**, 746.

Stevenson, A. C.—(1966) ‘Sex Chromatin and the Sex Ratio in Man’ in ‘The Sex Chromatin’. Edited by K. L. Moore. Philadelphia and London (W. B. Saunders & Co.).